# New genetic perspectives of the ambiguous pomfret as revealed by CR sequences

**DOI:** 10.3897/zookeys.719.19914

**Published:** 2017-12-07

**Authors:** Yuan Li, Yan Zhang, Longshan Lin, Tianxiang Gao, Liqin Liu

**Affiliations:** 1 Third Institute of Oceanography, State Oceanic Administration, Xiamen 361005, China; 2 Yellow Sea Fisheries Research Institute, Chinese Academy of Fishery Sciences, Qingdao 266071, China; 3 National Engineering Research Center for Marine Aquaculture, Zhejiang Ocean University, Zhoushan 316004, China

**Keywords:** Genetic structure, mitochondrial DNA, *Pampus argenteus*, population expansion, population genetics

## Abstract

*Pampus argenteus* is an economically important fish that is often erroneously identified as *Pampus echinogaster*. No population genetic analyses have been performed on the true *P. argenteus* species. Here, the mitochondrial control region (CR) was used to evaluate the population genetics and elaborate the historical demography of the Silver pomfret collected from six geographical locations in China, Pakistan, and Kuwait. A high level of genetic diversity was demonstrated in this species. Analysis of molecular variance (AMOVA) revealed that the genetic divergence was mainly derived from within the populations (*P* < 0.05). A historical demographic analysis indicated that the Silver pomfret experienced a recent population expansion during the late Pleistocene. The phylogeographical structure revealed two obvious lineages that diverged in the late Pleistocene, during which the Silver pomfret populations historically experienced exotic divergence and mixed again with differentiated populations. Currently, Silver pomfret populations have insufficient time to attain migration-drift equilibrium. Population genetic data of the Silver pomfret can provide preliminary genetic knowledge for its fishery management.

## Introduction

The Silver pomfret *Pampus argenteus* (Euphrasen, 1788) is an economically important species that plays a vital role in commercial fisheries (Divya et al. 2017). *Pampus argenteus* belongs to the family Stromateidae (Haedrich 1984; Yamada et al. 2009). Euphrasen (1788) provided a general morphological description of *P. argenteus* based on only one individual, and the original description did not include critical diagnostic characteristics that could be used to identify the species. Because of their high morphological similarity, *P. argenteus* and *Pampus echinogaster* (Basilewsky, 1855) are typically mistaken as the same species (Peng et al. 2010a, b; Zhao et al. 2010, 2011; Wu et al. 2012). However, the importance of the *Pampus* taxon has been recognized by Chinese researchers who have further studied *P. argenteus* and *P. echinogaster*. For instance, based on its morphological characteristics and DNA barcoding, Li et al. (2013) found that *P. argenteus* is distributed only in the southern waters of the Taiwan Strait, and the pomfret fishes inhabiting the Yellow Sea, Bohai Sea, and Northern East China Sea were not *P. argenteus*. Sun (2015) reported significant genetic differences between “*P. argenteus*” farmed in Kuwait and China, suggesting that the Silver pomfret farmed in China may be *P. echinogaster* rather than *P. argenteus*. Additionally, Liu et al. (2015) asserted that the Yellow Sea and Bohai Sea harbored only *P. echinogaster* and *Pampus punctatissimus* (Temminck & Schlegel, 1845) and that *P. argenteus* was absent from these regions. Finally, Li et al. (2017) proposed the diagnostic characteristics of *P. echinogaster*, which were significantly different from those of *P. argenteus*.

Indeed, numerous studies have shown that *P. argenteus* is absent from the Yellow Sea, Bohai Sea, and Northern East China Sea and that the so-called “*P. argenteus*” referenced in previous studies (Meng et al. 2009; Peng et al. 2010a, b; Zhao et al. 2010, 2011; Wu et al. 2012) is actually *P. echinogaster*. Therefore, we know that the Silver pomfret is a warm-water species that is widely distributed south of the Taiwan Strait and across Indonesia to the Persian Gulf (Yamada et al. 2009; Li et al. 2013). After measuring morphological characteristics of many specimen, the major morphological diagnostic characteristics of *P. argenteus* can be summarized as follows: oval body; dorsal fin VII-VIII-39-43, pectoral fin 21–29, anal fin V-VI-35-41, caudal fin 26–28; transverse occipital canals and dorsal branches of the lateral-line canal on top of the head with a shallow arc-like rear edge; ventral branches slightly longer than the dorsal branches, extending backward and not reaching the base of the dorsal fin, with an eyebrow-like shape; gill rakers thin, sparse, 2-3+8-9=10-12; and vertebrae 37–38.

To date, no population genetic analyses have been reported based on the true *P. argenteus* species. Therefore, one objective of the present study is to investigate the true population genetics of the Silver pomfret to attract the attention of relevant researchers. Another objective is to elucidate the historical population dynamics of this species at the mitochondrial level for the first time. Analyzing mitochondrial DNA is an effective method for detecting population genetic structure and diversity based on haploid or maternally inherited genes or genes that are not subject to recombination (Engelbrecht et al. 2000). In this study, six populations of Silver pomfret were collected from the coastal waters in Kuwait, Pakistan, and China, and the sequences of the mitochondrial DNA control region (CR) were analyzed.

## Materials and methods

### Sample collection

**Figure 1.**
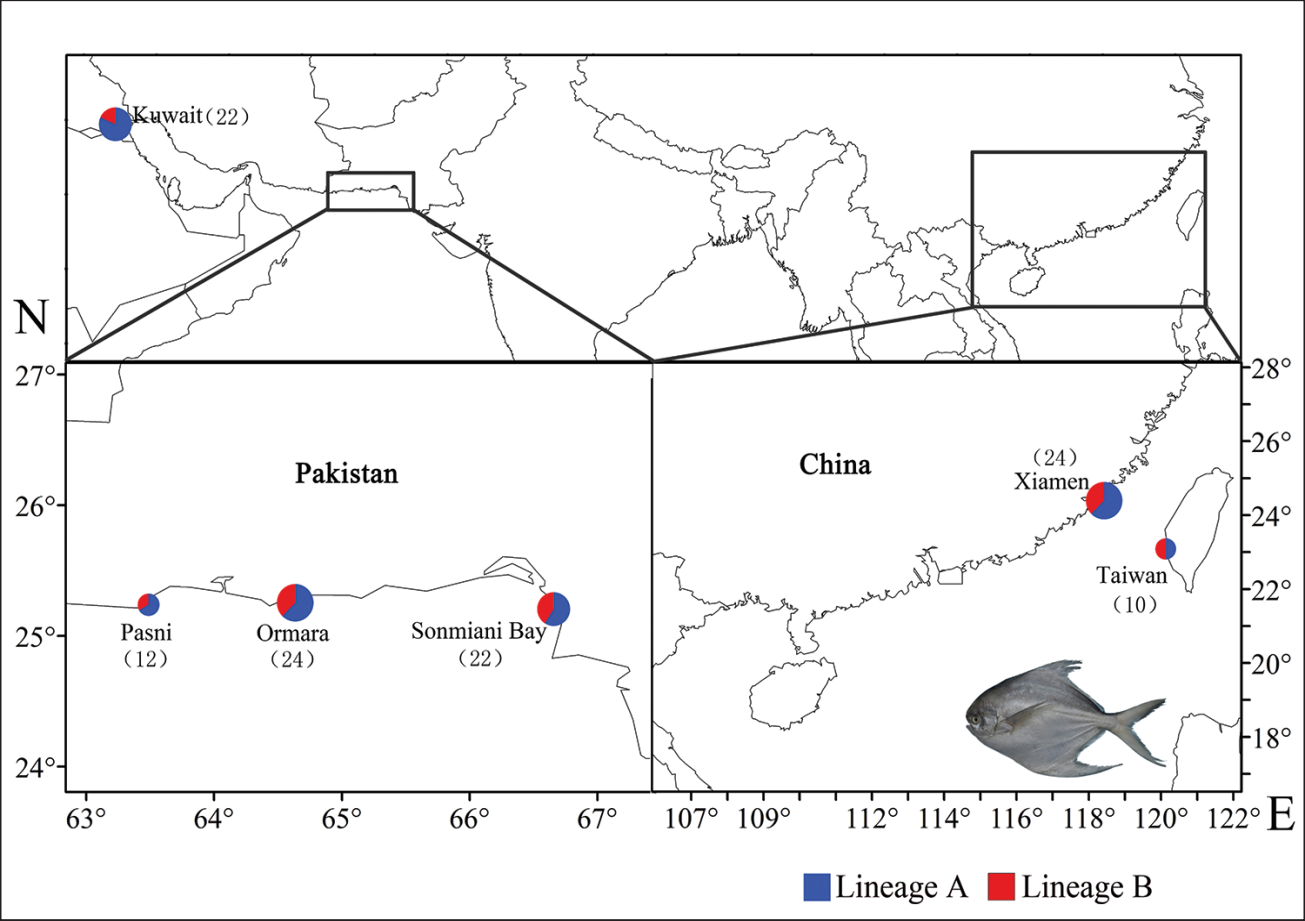
Sampling locations of *P. argenteus*.

In total, 114 Silver pomfret individuals were collected from the northern waters in Kuwait, Sonmiani Bay, Ormara, Pasni, Xiamen, and Taiwan between 2010 and 2014 (Figure 1). All individuals were identified according to their morphological characteristics (Li et al. 2013) to ensure the accuracy of the species identification. Then, the back-muscle tissues were excised and preserved in 95% alcohol for the subsequent experiments.

### DNA extraction, amplification and sequencing

Genomic DNA was isolated from muscle tissue by proteinase *K* digestion and extracted with Qiagen DNeasy kit. The extracted DNA was assessed by 1.5% agarose gel electrophoresis and stored at –20 °C for PCR amplification. The mtDNA CR was amplified with the primers F-gao: 5'-GAAGTTAAAATCTTCCCTTTTGC-3' (forward), and R-gao: 5'-GGCCCTGAAGTAGGAACCAAA-3' (reverse). Each PCR was performed in a 25 μL reaction mixture containing 17.5 μL of ultrapure water, 2.5 μL of 10× PCR buffer, 2 μL of dNTPs, 1 μL of each primer (5 μM), 0.15 μL of Taq polymerase, and 1 μL of DNA template. PCR amplification was performed in a Biometra thermal cycler under the following conditions: 5 min of initial denaturation at 95 °C; 30 cycles of 45 s at 94 °C for denaturation, 45 s at 50 °C for annealing, and 45 s at 72 °C for extension; and a final extension at 72 °C for 10 min. PCR products were purified, and both strands were sequenced. The newly isolated nucleotide sequences were deposited in GenBank under accession numbers MF402948–MF402998. Two CR sequences of *Pampus chinensis* (Euphrasen, 1788) were used as the out-group.

### Data analysis

CR sequences were edited and aligned using DNASTAR software. Polymorphic sites, haplotype number, and molecular diversity indices for each population were calculated using ARLEQUIN version 3.5 (Excoffier et al. 2005). Genetic relationships among haplotypes were reconstructed using the neighbor-joining (NJ) method implemented with 1000 replicates in MEGA 5.0 (Tamura et al. 2011). Analysis of molecular variation (AMOVA) was performed using ARLEQUIN to investigate the partition of genetic variation among the populations. An unrooted minimum spanning tree (MST) was constructed via the MINSPNET algorithm as implemented in ARLEQUIN to show the relationship among haplotypes and subsequently drawn by hand (Excoffier et al. 2005). Historical demography/spatial expansions were inferred by neutrality testing and mismatch distribution analysis, as implemented in ARLEQUIN. Deviations from neutrality were evaluated using Fu’s *F*_S_ and Tajima’s *D.* Nucleotide mismatch distributions were applied to assess population growth and spatial range expansion. A molecular clock-based time estimate provided an approximate timeframe for evaluating phylogeographical hypotheses. Historical demographic expansions were also investigated by the examination of frequency distributions of pair-wise differences between sequences (mismatch distribution), based on three parameters: *θ_0_, θ_1_* (*θ* before and after population growth) and *τ* (time since expansion, expressed in units of mutational time) (Rogers and Harpending 1992). The values of *τ* were transformed to estimates of real time since expansion with the equation *τ*=2×*μ*×*t* where *μ* is the mutation rate for the whole sequence under study and *t* is the time since expansion. In the present study, a sequence divergence rate of 0.5–1× 10^−7^/site/year was applied to the CR sequences of *P. argenteus* (Bowen et al. 2001). Bayesian skyline plots were created with BEAST v.8 (Drummond and Rambaut 2007). In the present study, a sequence divergence rate of 5%–10% /Myr (Bowen et al. 2001) was applied to the CR sequences of *P. argenteus*.

## Results

### Genetic diversity

After a manual correction, the CR fragment sequences were 450~453 bp in length, including a 70-bp partial fragment of the tRNA^pro^, and no variable site was detected in the tRNA fragment. After deleting the 70-bp fragment, the obtained target fragment was 380~383 bp in length, which corresponded to the 15,699–16,080 bp region of the complete mitogenome of *P. argenteus* (KJ569773). Thirty-eight variable sites and 23 parsimony-informative sites were assessed within the target fragment. There were 23 transitions, five transversions, and five insertions/deletions. The ratio of transitions to transversions was 4.6, indicating that the mutations in the CR sequence of *P. argenteus* had not reached saturation. The A+T content (70.33%) was significantly higher than the G+C content, indicating a significant AT preference.

All variable sites defined 51 haplotypes among the 114 individuals. No haplotype was shared between the six populations, and forty-two specific haplotypes were detected among all individuals, accounting for 82.4% of the total haplotypes (Table 1). The population from Ormara exhibited the most specific haplotypes (10), whereas the population from Taiwan exhibited the fewest specific haplotypes (4). Hap_27 was the dominant haplotype and was shared by 22 individuals (Table 2). High levels of haplotype diversity (*h)* were detected within each population, demonstrating a high level of genetic diversity in this species. In contrast, low levels of nucleotide diversity (*π*) were observed. Overall, the average values of the *h* and *π* were 0.932 ± 0.013 and 0.018 ± 0.010, respectively (Table 1).

**Table 1.** Information and molecular indices of *P. argenteus*.

ID	Populations	Number	Date	NH	NUH	*h* ± SD	*л*± SD	*k* ± SD
S	Sonmiani Bay	22	2010.12	12	7	0.8658±0.0652	0.0116±0.0066	4.4416±2.2765
N	Pasni	12	2010.12	10	6	0.9697±0.0443	0.0169±0.0097	6.4394±3.2798
O	Ormara	24	2010.12	15	10	0.9275±0.0388	0.0146±0.0081	5.5906±2.7812
K	Kuwait	22	2011.09	10	8	0.7100±0.1064	0.0114±0.0065	4.3593±2.2397
T	Taiwan	10	2012.09	8	4	0.9333±0.0773	0.0114±0.0070	4.3778±2.3612
X	Xiamen	24	2014.04	13	8	0.9203±0.0326	0.0108±0.0062	4.1051±2.1186
Total		114	–	51	–	0.9322±0.0134	0.0183±0.0096	7.0202±3.3217

Note: NH, numbers of haplotypes; NUH, numbers of specific haplotypes; *h*, haplotype diversity; *π*, nucleotide diversity; *k*, average number of pairwise differences.

### Genetic structure

**Figure 2.**
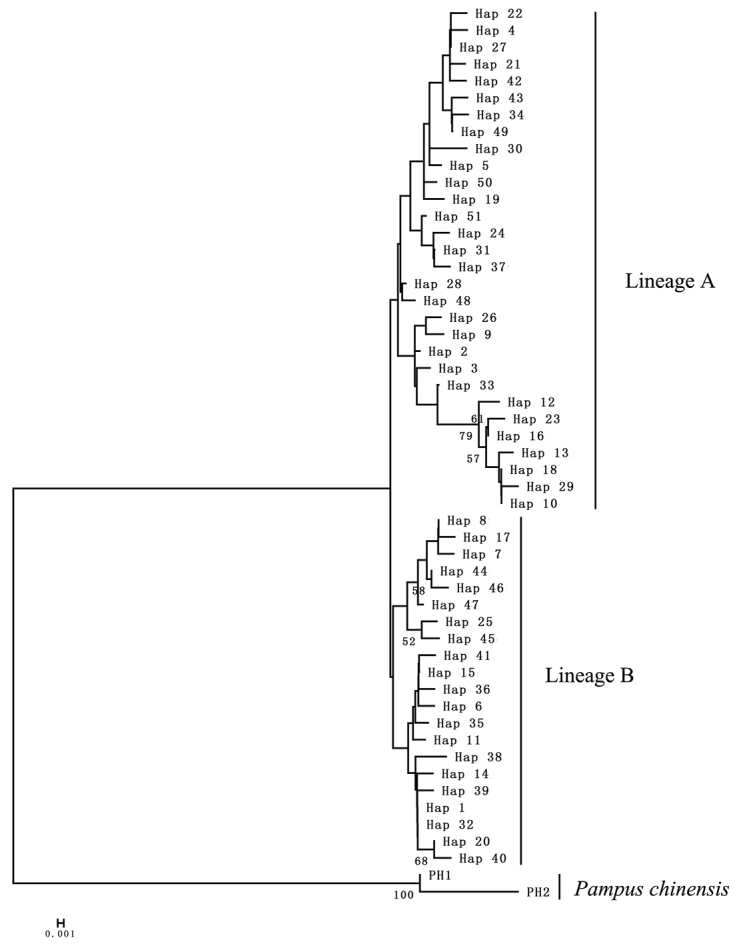
NJ tree of CR haplotypes of *P. argenteus*. *Pampus chinensis* was used as the out-group. Bootstrap supports >50 in 1,000 replicates are shown.

**Figure 3.**
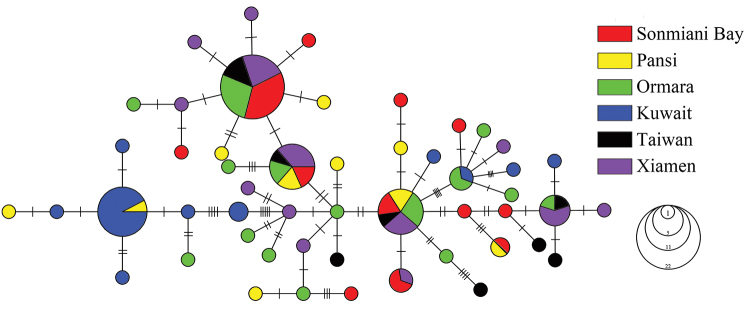
Unrooted minimum spanning tree depicting the genetic relationship among the CR haplotypes of *P. argenteus*. Circle sizes are proportional to the haplotype frequency. Perpendicular tick marks on the lines joining the haplotypes represent the number of nucleotide substitutions.

A NJ tree was constructed based on the 51 CR haplotypes using *P. chinensis* as out-group, and two deeply divergent lineages were identified in the six populations that were not geographically concordant (Figure 2). Lineage A comprised 30 haplotypes (74 individuals), whereas lineage B comprised 21 haplotypes (40 individuals). No significant differences were observed in the haplotype distribution of the two lineages, and both lineages were found in all populations (Table 2, Figure 2). Lineage A obviously dominated the population from Kuwait (81.82%), followed by the population from Pakistan (62.07%). However, lineage B dominated the Chinese populations (41.18%) compared with its presence in the Arabian Sea populations (32.5%). The frequency of lineage A in each population was reduced from 81.82% (Kuwait) to 50% (Taiwan). Two shallow lineages were also detected in MST among the 51 CR haplotypes (Figure 3). There were no significant differences in the distribution of the haplotypes in both lineages other than the specific ones mentioned above (Table 2).

**Table 2.** Distribution of haplotypes among all silver pomfret populations in lineage A and B.

	haplotype	Total	S	N	O	K	T	X		haplotype	Total	S	N	O	K	T	X
Lineage A	Hap_2	1						1	Lineage B	Hap_1	11	2	2	3		1	3
	Hap_3	1						1		Hap_6	1						1
	Hap_4	1						1		Hap_7	1						1
	Hap_5	11	2	2	2		1	4		Hap_8	5			1		1	3
	Hap_9	2				2				Hap_11	1				1		
	Hap_10	13		1		12				Hap_14	1				1		
	Hap_12	1				1				Hap_15	3			2	1		
	Hap_13	1				1				Hap_17	1				1		
	Hap_16	1				1				Hap_20	1		1				
	Hap_18	1				1				Hap_25	2	1	1				
	Hap_19	1		1						Hap_32	1			1			
	Hap_21	1		1						Hap_35	1			1			
	Hap_22	1		1						Hap_36	1			1			
	Hap_23	1		1						Hap_38	1	1					
	Hap_24	1		1						Hap_39	3	2					1
	Hap_26	1			1					Hap_40	1	1					
	Hap_27	22	8		6		3	5		Hap_41	1	1					
	Hap_28	1			1					Hap_44	1	1					
	Hap_29	1			1					Hap_45	1					1	
	Hap_30	1			1					Hap_46	1					1	
	Hap_31	1			1					Hap_47	1					1	
	Hap_33	1			1				Total		40	9	4	9	4	5	9
	Hap_34	1			1												
	Hap_37	1	1														
	Hap_42	1	1														
	Hap_43	1	1														
	Hap_48	1					1										
	Hap_49	1						1									
	Hap_50	1						1									
	Hap_51	1						1									
Total		74	13	8	15	18	5	15									

Based on the best model, i.e., TrN+G, the net genetic distance between lineage A and lineage B was 0.0058. Based on the 5–10%/MY (million years) divergence rate, the time since the population divergence occurred was estimated to be 0.06–0.12 million years ago, dating back to the late Pleistocene.

The *F*_ST_ values between six populations were low (from 0.012 to 0.062) and statistically non-significant, except for those from the Xiamen and Kuwait populations (Table 3). All results showed that genetic differentiation was not significant between different populations. The negative *F*_ST_ values suggested that the genetic differentiation among individuals was higher than that within populations. AMOVA revealed that the variability among the samples yielded an *F*_ST_= 0.3328 (*P*<0.001) as one gene pool, and the divergence was attributable to 66.72% of the genetic variation among the populations. To further investigate the possible effects, the populations were partitioned into two and three gene pools, and both pools yielded a significant divergence within the populations (*P* < 0.001) (Table 4). Therefore, the divergence among the populations was very weak with no statistical significance, whereas the genetic divergence was mainly derived from within the populations with statistical significance. Thus, in all cases, no significant genetic structure was identified across the entire geographical sampling range of the Silver pomfret.

**Table 3.** Matrix of pairwise *F*_ST_ values between six *P. argenteus* populations based on mitochondrial CR sequences.

	X	K	N	O	S	T
X						
K	0.061*					
N	0.012	0.046				
O	0.018	0.051	-0.013			
S	0.014	0.062	0.018	0.015		
T	-0.025	0.061	0.019	0.044	0.022	

* significant at *P* < 0.05 by the permutation test.

**Table 4.** AMOVA of *P. argenteus* populations based on mitochondrial CR sequences.

**Source of variation**	**Sum of squares**	**Percentage**	***F* statistic**	***P***
**One gene pool**				
Among populations	128.103	33.28	*F*_ST_= 0.3328	0.000
Within populations	268.537	66.72		
**Two gene pools** (K, S, O, N) (X, T)				
Among groups	20.768	-5.02	*F*_CT_= -0.05025	0.608
Among populations within groups	107.336	36.67	*F*_SC_= 0.34914	0.000
Within populations	268.537	68.36	*F*_ST_= 0.31643	0.000
**Three gene pools** (K) (S, O, N) (X, T)				
Among groups	120.461	39.79	*F*_CT_=0.39786	0.054
Among populations within groups	7.642	0.09	*F*_SC_=0.00143	0.425
Within populations	268.537	60.13	*F*_ST_=0.39872	0.000

### Historical demographics

**Figure 4.**
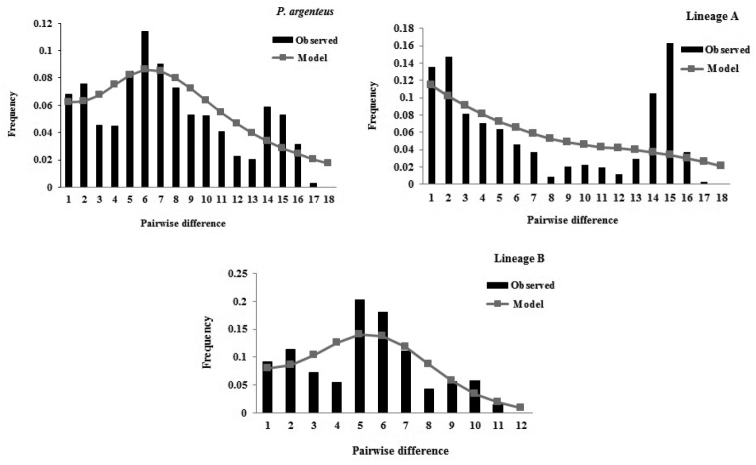
Mismatch distributions of control region haplotypes of *P. argenteus*.

The observed mismatch distributions of Silver pomfret were established for the two lineages (Figure 4). The mismatch distribution of lineage B exhibited a unimodal pattern that was consistent with the expected distribution in the population expansion model. In contrast, the mismatch distribution of lineage A was bimodal with two peaks. The *F*_S_ test was significantly negative for both lineages (*P* < 0.05), whereas the *D* test was not significantly negative (*P* > 0.05). However, both the *SSD* and *HRI* tests were not statistically significant (*P* > 0.05), suggesting that the two lineages were consistent with the null hypothesis of the sudden expansion model. Thus, the studied Silver pomfret populations experienced a recent expansion event.

The peak τ of the nucleotide mismatch distribution provides information that can be utilized to estimate the approximate time of the population expansion. In this study, the τ values of lineages A and B were 14.889 and 5.426, respectively (Table 5). Therefore, the time since the expansion of lineages A and B was estimated to be 3.9 × 10^5^–7.8 × 10^5^ and 1.4 × 10^5^–2.8 × 10^5^ years ago based on the divergence rate of the mitochondrial CR (5–10%/MY) and τ values, respectively. The ratio values (*θ_1_*/*θ_0_*), which estimated the effective female population sizes after and before the expansion, were 2000 for lineage A and infinite for lineage B (Table 5).

**Table 5.** Summary of molecular diversity, neutral test and goodness-of-fit test for *P. argenteus*.

	Number	NH	*h* ± SD	*л*± SD	*k* ± SD	Tajima’s *D*		Fu’s *Fs*		Goodness-of-fit test				
						*D*	*P*	*Fs*	*P*	*τ*	*θ_0_*	*θ_1_*	*SSD*	*HRI*
Lineage A	74	30	0.865±0.028	0.017±0.009	6.557±3.134	-0.046	0.552	-8.967	0.017	14.889	0.004	8.235	0.031ns	0.033ns
Lineage B	40	21	0.908±0.034	0.011±0.006	4.222±2.140	-1.261	0.080	-9.606	0.001	5.426	0.000	11.953	0.015ns	0.037ns
All	114	51	0.932±0.013	0.018±0.010	7.020±3.322	-0.094	0.058	-2.454	0.049	10.555	0.000	7.200	0.038ns	0.042ns

Note: NH, numbers of haplotypes; *h*, haplotype diversity; *π*, nucleotide diversity; *k*, average number of pairwise differences; ns, *P*>0.05.

**Figure 5.**
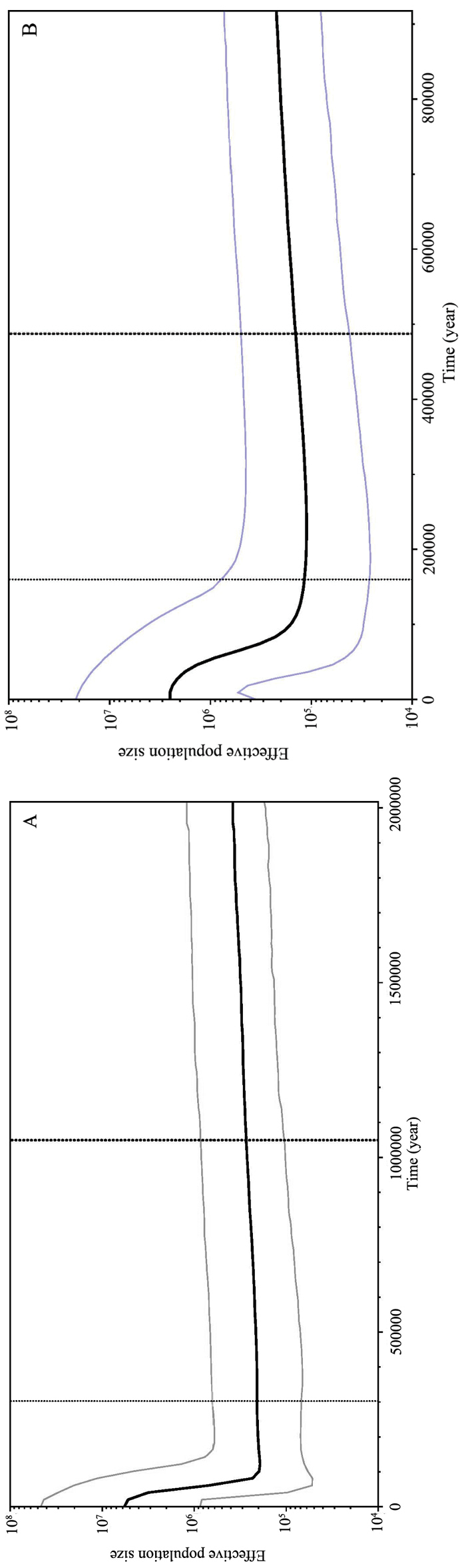
Bayesian skyline plots showing *N_ef_*T (*N_ef_* = effective population size; T = generation time) changes over time for *P. argenteus* based on CR sequences. The upper and lower limits of the blue line represent the 95% confidence intervals of highest posterior densities (HPD) analysis. The black line represents median estimates of *N_ef_*T.

The Bayesian skyline plots revealed a detailed demographic history of population size changes, from which we could see that both lineages A and B had undergone population expansion in the late Pleistocene. The effective population size of lineage A increased slowly after the last glacial maximum (LGM) approximately 3.2 × 10^5^ years before the present, and the effective population size of lineage B increased sharply from 1.7 × 10^5^ years ago (Figure 5).

## Discussion

No population genetic studies to elucidate the true population genetics of *P. argenteus* have been reported. Thus, understanding the genetic background of this species is of great theoretical and practical value for the conservation of its genetic diversity and sustainable resource utilization.

### Genetic diversity

The genetic diversity in species is a result of the long-term evolution of organisms, and the level of genetic diversity is closely related to the survival and evolutionary potential of the species, of which *h* and *π* are two important indicators. In this study, high *h* and low *π* were detected in six *P. argenteus* populations, and the results supported the second population rapid growth hypothesis of marine fishes as interpreted by Grant and Bowen (1998).

Currently, the high diversity in this species may be related to the following aspects. First, this species has an extensive distribution area and varying habits. Silver pomfret are found from the Taiwan Strait to the Indian Ocean. This long coastline has created diverse marine ecological environments in which this species is successfully adaptive to local habitat conditions. Second, this species has numerous effective populations. Despite a declining trend in the amount of pomfret resources, numerous recruitment populations are available to ensure an effective population, which was evaluated by acoustic fishery resources (Jia et al. 2004). Third, this species has advantages due to its geographical distribution. Since the Quaternary, substantial increases and decreases in global temperature have resulted in a decreased genetic diversity, and the most seriously affected species are located at the southern and northern edges of the distribution area (Hewitt 1996). The distribution area of *P. argenteus* occurs in a relatively central location that is less affected by global glacial climate fluctuations; therefore, a relatively high genetic diversity may more easily occur.

### Genetic structure and historical demographics

Two lineages were tested using a NJ tree and MST based on all haplotypes. The spatial variation in the haplotype frequencies between the two lineages was absent, indicating a high degree of genetic homogeneity among the six populations. The genetic structure may be a result of both historical and contemporary processes. All population structure analyses were concordant with the null hypothesis of panmixia despite the well-defined phylogeographical structures of the mtDNA haplotypes. Numerous studies have confirmed that the phylogeographical patterns and population genetic structures of marine species are related to specific geological events or environmental factors, and the isolation due to Pleistocene glaciation is likely the main reason for the genetic differentiation of species (Liu et al. 2007; Shen et al. 2011; Han et al. 2012; Qiu et al. 2016). Therefore, we hypothesized that the phylogeographical structure of the Silver pomfret was connected to a second admixture in which its populations historically experienced exotic divergence and mixed again with differentiated populations (Avise 2000). The biomass of marine organisms decreases sharply with periodic climate fluctuations. In addition, few surviving individuals remained in the limited shelters during the LGM. Due to the rising sea levels that occurred after the last glacial period, the Silver pomfret potentially underwent a re-colonization event, and the effective maternal population size grew rapidly. The derivative populations that experienced population isolation were connected to the second admixture, which eliminated the partial genetic divergence that previously accumulated. In this case, the genetic differentiation that was detected across the distribution range of the Silver pomfret was compatible with the hypothesis of recent range expansion and insufficient time to attain migration-drift equilibrium (Slatkin 1993), which was considered the most important factor.

In addition to historical events, contemporary factors, including oceanic currents and life history characteristics of the species, are important factors that affect the genetic structure of species in marine environments. Similar to numerous marine pelagic fishes, the Silver pomfret exhibits a highly migratory behavior and large population size and dispersal potential during the early life stage, which could lead to frequent gene flow among different populations (Ward et al. 1994). In general, ocean currents play an important role in transporting the larvae of marine organisms, which could allow substantial dispersal and high connectivity among different populations (Liu et al. 2007). Thus, the Silver pomfret was hypothesized to travel a long range, given its larval stage and the current velocity near the coasts of China and Pakistan, which could sufficiently explain the low level of genetic divergence in this species.

Unfortunately, we only collected six geographical populations of the Silver pomfret, which is not enough for an even sampling throughout its entire distribution in the Indo–Pacific Ocean. The population genetics of this species may be one-sided in this study and remain to be discussed further. Therefore, the Silver pomfret samples of an intermediate distribution need to be collected and will further verify our results.

### Conservation implications for fishery management

It is necessary to assess the genetic population diversity and genetic structure of marine fish for fisheries management and conservation. The contemporary genetic structure of the Silver pomfret revealed in this study can preliminarily improve genetic knowledge and provide a firm basis of fishery stocks in the Indo-Pacific Oceans. Although the Silver pomfret currently exhibits a relatively high genetic diversity, it is likely to experience a disaster similar to that experienced by traditional economic fish, e.g., *Larimichthys polyactis* (Bleeker, 1877), *L. crocea* (Richardson, 1846) and *Trichiurus haumela* (Forsskål, 1775), if attention is not paid to the conservation of resources. In fact, a decline in the Silver pomfret resources has been reported in some waters due to over-fishing and the devastation of marine ecology. Therefore, fishery management measures regarding the Silver pomfret must be implemented in a timely manner.
